# Case I. Typhoid Fever—Strychnia, Etc.

**Published:** 1871-02

**Authors:** 


### Clinical Reports.

### CLINICAL CASES IN MEDICAL WARDS OF MERCY HOSPITAL.

Clinic by PROF. DAVIS. From Notes by S.

MERCY HOSPITAL, *January, 1870.*

CASE I.—TYPHOID FEVER—STRYCHNIA, ETC.—A. B., aged 27 years; native of Ireland; laborer; was admitted into the Hospital eight days since with all the symptoms of a severe form of enteric typhoid fever, then in the middle of the second week of its progress. The tongue was dry and brown, with redness of the tip and edges; the abdomen tympanitic; face suffused with redness; pulse 120 per minute; skin moderately hot and dry; intestinal evacuations eight or ten times in the twenty-four hours, thin and dark brown; urine less than natural; respirations short and twenty per minute, with some cough and harsh respiratory murmur in the upper and anterior part of the chest, and deficient murmur with dullness in the lower part of the left side. Mind dull, but not much wandering.

To arrest the progress of the abdominal lesions, and also aid in giving tone to the capillary circulation generally, he was put upon moderate doses of oil of turpentine and tincture of opium in the form of an emulsion, every three hours; and to counteract the tendency to pulmonary congestion, he took one teaspoonful of the following prescription three times a-day:

℞ Hydrochlorate Ammonia, ʒiii.

Tart. Ant. et Pot., 2 grs.

Sulp. Morph., 3 grs.

Syrup Liquorice, ℥iv.

M.

His nourishment, administered regularly, consisted of sweet milk and wheat-flour porridge, alternated with beef-tea. During the first five days of this treatment, all the important symptoms improved. The temperature diminished; the cough ceased; the pulse gradually fell to 100; abdominal tympanites diminished; and the intestinal discharges were reduced in frequency to two in the twenty-four hours. But the tongue remained dry and the mental faculties dull. On account of the diminished frequency of discharges, the emulsion was given only every four hours; otherwise the treatment was not altered. During the sixth and seventh days after admission, which would be about the middle of the third week after the patient had taken to the bed, the symptoms rapidly assumed a more unfavorable aspect. The pulse increased to 130 per minute, and was quick, soft, and weak; the mind more wandering; and the intestinal evacuations more frequent and partially involuntary. (The attention of the class was called particularly to the state of the pulse and intestinal discharges as indicating a dangerous degree of depression of the functions of the nervous system, especially that part of it denominated "excito-motory" by Marshall Hall; and the necessity of some treatment for promptly arresting further change in that direction.) The Professor stated that he knew of no remedy as direct and efficient for sustaining the nervous functions of organic and animal life as strychnia.

When the muscular contractions of the heart became weak, and the sphincter muscles gave indications of relaxation in the advanced stage of typhoid fever, he had for many years resorted to the use of strychnia with great advantage. He generally gave it in connection with nitric acid, and when the bowels are moving frequently, added also tincture of opium. For this patient he ordered the following formulæ:

℞ Strychnia, 1 gr.

Nitric acid, ʒi.

Tinct. Opii., ʒiii.

Water, ℥iii.

Of which he directed one teaspoonful to be given every four hours, and the turpentine and laudanum emulsion to be given between. Milk porridge and beef-tea were continued for nourishment as before.

About five days later the attention of the class was again called to this patient, and he presented all the symptoms of commencing convalesence. This treatment had been continued without any other change than to lengthen the intervals between the doses of his medicine.

